# Unmeasured Osmoles: The Hidden Solutes Obscuring a Hyponatremia Evaluation

**DOI:** 10.7759/cureus.66833

**Published:** 2024-08-14

**Authors:** Jay Mathias, Shachi Lovekar, Jayson Yap

**Affiliations:** 1 Internal Medicine, Wright State University, Dayton, USA; 2 Nephrology, Dayton Veterans Affairs (VA) Medical Center, Dayton, USA

**Keywords:** ethanol, hyperosmolar hyponatremia, urine osmolality, unmeasured osmoles, serum osmolality

## Abstract

Hyponatremia is defined as serum sodium less than 135 mEq/L and is principally a result of water excess relative to total body sodium content. The evaluation of hyponatremia is incomplete without a careful assessment of the patient’s volume status, history, and acquisition of both serum and urine osmolality and sodium studies. Many of these studies can be affected by various clinical factors, and these nuances should be considered while interpreting the results. This is because these results guide the etiologic diagnosis of hyponatremia and consequently its management. In this report, we describe a 50-year-old male being evaluated for hyponatremia found to have unusual serum/urine osmolality studies but ultimately found to have an unmeasured serum osmole (ethanol) interfering with the interpretation of these results. Clinical scenarios that interfere with serum and urine studies commonly obtained in a hyponatremia evaluation are reviewed and an equation to correct for ethanol's osmotic contribution is described.

## Introduction

Hyponatremia is defined as serum sodium less than 135 mEq/L and is principally a result of water excess relative to total body sodium content [1,2]. For simplicity, hyponatremia can result from too much water intake, too little water excretion, or a combination of both. Water excretion by the kidney is regulated by antidiuretic hormone (ADH), specifically, an abundance of ADH restricts water excretion. The kidney can also be limited in its ability to excrete free water if there is an intrinsic diluting defect of the kidney or inadequate solute intake, which hampers free water excretion by the kidney, despite an intact diluting mechanism. The evaluation of hyponatremia is incomplete without a careful assessment of the patient’s volume status and history. Additionally, serum and urine studies help in further characterizing the etiology of hyponatremia into categories based on osmolality (hyperosmolar, iso-osmolar, and hypo-osmolar) which impact the treatment of hyponatremia [1]. Many of these studies can be impacted by various clinical factors and should be considered while interpreting the results as these results guide the etiologic diagnosis of hyponatremia and consequently its management [1]. One clinical scenario exemplifying this is the presence of ethanol in the serum and urine which influences osmolality results. We present a case of a patient being evaluated for chronic hyponatremia with unusual serum/urine studies but ultimately found to have an unmeasured serum osmole (ethanol) interfering with the interpretation of these results.

## Case presentation

A 50-year-old male with a past medical history of post-traumatic stress disorder, hypertension, benign prostatic hyperplasia, and alcohol-use disorder (intake of six 12 oz beers daily for years) was evaluated by the nephrology service for persistent hyponatremia noted on routine labwork for four months. For multiple visits over several months, he was consistently clinically euvolemic and attentive and did not display signs of alcohol intoxication. Additionally, the patient reported on multiple clinical visits that he remained abstinent from alcohol. His laboratory workup yielded serum sodium levels ranging between 131 to 134 mEq/L with concurrent urine osmolalities ranging from 162 to 254 mOsm/kg, and urine sodium ranging between 10 to 30 mEq/L. Notably, concurrent serum osmolality values were consistently above 295 mOsm/kg, and he had a consistently elevated osmolar gap (ranging between 24 to 64 mOsm/kg). At this point, the differential diagnosis included hyperosmolar hyponatremia causes such as hyperglycemia, but less likely to be mannitol or contrast dye due to lack of clinical history supporting these etiologies. Additionally, diagnoses such as thyroid abnormalities, adrenal abnormalities, syndrome of inappropriate ADH secretion (SIADH), and beer potomania were considered. To further evaluate the etiology of the patient's hyponatremia, labwork including renal function, thyroid function, lipid profile, and glucose levels were obtained and were all within normal limits. There was no significant alteration to his bicarbonate, nor an elevated anion gap, reducing the likelihood of lactic acidemia or keto-acidemia. Furthermore, a serum protein electrophoresis was obtained which did not support paraproteinemia. At this point, etiologies such as thyroid and adrenal abnormalities were not felt to be contributing to the patient's hyponatremia due to a lack of clinical features and supporting labwork. Additionally, SIADH was felt to be unlikely due to his urine not being significantly concentrated. To further investigate his elevated osmolar gap, blood tests to ascertain levels of several unmeasured osmoles such as acetone, methanol, and isopropanol were obtained, and these were not detected. A blood ethanol level was obtained and was elevated to 115 mg/dL with a corresponding urine drug screen positive only for ethanol. The presence of ethanol was believed to be interfering with the patient’s osmolality studies (serum/urine); and after adjusting for ethanol’s osmotic contribution, the serum osmolality and osmolar gap corrected to anticipated values for a true hyponatremia. Thus, we were able to confidently diagnose him with beer potomania and recommend alcohol abstinence for management. A summary of the urine and serum lab studies can be found in Table 1.

**Table 1:** Lab values and impact of applying alcohol correction to the calculated and measured serum osmolality in our patient. ^a ^Before applying ethanol correction. ^b ^After applying ethanol correction.

Laboratory value (normal range)	Value
Sodium (136-145)	129 mEq/L
Blood urea nitrogen (6-23)	5 mg/dL
Glucose (70-100)	93 mg/dL
Measured serum osmolality^a^ (281-303)	304 mOsm/kg
Calculated serum osmolality^a^	264 mOsm/kg
Urine osmolality (50-1400)	254 mOsm/kg
Urine sodium	31 mmol/L
Osmolar gap^a^	40 mOsm/kg
Ethanol	115 mg/dL
Measured serum osmolality^b^	279 mOsm/kg
Calculated serum osmolality^b^	289 mOsm/kg
Osmolar gap^b^	15
Urine drug screen	Positive for ethanol
Aspartate aminotransferase (8-33)	52 U/L
Alanine transaminase (7-56)	101 U/L
Albumin (3.5-5.5)	4.6 g/L
Total bilirubin (0.1-1.2)	0.5 mg/dL
Alkaline phosphatase (44-147)	93 IU/L

## Discussion

Hyponatremia is a common electrolyte abnormality affecting roughly 5% of adults and is encountered in 35% of hospitalized patients [1]. An objective etiologic evaluation of hyponatremia involves an assessment of the patient’s volume status along with additional serum and urine studies. Serum and urine studies commonly obtained in the evaluation of hyponatremia include serum sodium and osmolality along with urine sodium and osmolality. Many of these tests can be compromised by (1) the laboratory techniques used to measure these values, (2) the presence of osmotically active substances in the plasma, and (3) the presence of unmeasured osmoles (as in our patient). When taken at face value, the results of these tests can lead the interpreting physician to a wrong etiology of hyponatremia and inappropriate treatments.

As mentioned, the laboratory techniques used to measure serum electrolytes and osmolality can be an area of manipulation and misinterpretation. Today, the use of ion-selective electrodes (ISE) has become the mainstay of measuring serum electrolytes. This technique involves the use of two electrodes, one being exposed to a reference sample and another electrode being exposed to the patient sample. The device detects a potential difference between the patient sample and the reference solution which is logarithmically proportional to the serum concentration of the ion of interest [3,4]. There are two types of ISE used for serum electrolyte measurements, indirect ISE and direct ISE. Indirect ISE, used in auto-analyzers, as at our institution, are more commonly used in patient routine lab work in larger laboratories, whereas direct ISE are used in point-of-care based equipment at the bedside [3]. The known limitation of indirect ISE when measuring serum sodium is the “electrolyte exclusion effect.” To understand this, recall that serum is composed of solid (7%) and aqueous (93%) phases. Expansion of the solid phase, e.g., the presence of abnormally elevated lipids or proteins, is accompanied by a reciprocal reduction in the aqueous phase per volume of plasma. Sodium is present exclusively in the aqueous phase of serum (or serum water content). While both indirect and direct ISE measure sodium content in the water fraction of serum; the indirect ISE requires dilution of an exact volume of the test sample with a fixed volume of standardized diluent. In contrast, the direct ISE measures sodium level in an undiluted serum sample and gives accurate results even if the volume of the serum sample is modified. When the aqueous phase of serum is reduced due to expansion of the solid phase as noted above, the dilution of the exact volume of the test sample in indirect ISE leads to a higher dilution effect than intended as it does not take into account the serum water content. This leads to erroneously low results of serum sodium and is thus classified as pseudohyponatremia [5-7]. Serum and urine osmolality are commonly measured in the lab using freezing point depression (FPD). In general, the laboratory technique to determine the osmolality of a solution determines the freezing point of that solution and can calculate the osmolality of the solution based on the magnitude of change of the freezing point [8]. The few limitations of this technique can often be avoided by proper centrifugation prior to testing to remove particulate matter, cleaning of sample containers after a test to avoid interference from a prior sample, and removal of air bubbles [8].

Furthermore, the measured serum sodium can be lowered by the presence of osmotically active substances (often termed effective osmoles), such as glucose, mannitol, and contrast, in the serum. Effective osmoles are called such due to their inability to readily cross the cell membrane and thus contribute to an osmotic gradient. Before moving forward, it is important to recall the difference between osmolality and tonicity. Osmolality is the total number of osmoles, regardless of whether they are osmotically active or not, in a kilogram of solvent and this is what is measured in the serum osmolality test. In contrast, the tonicity of a solution refers specifically to the osmotically active osmoles in the solvent, and there is no direct measurement for tonicity. Osmotically active molecules when present in the serum drag intracellular water into the extracellular space down an osmotic gradient producing a dilutional effect and true hyponatremia. Removal of the osmotically active molecule from the serum usually resolves the hyponatremia as seen in hyperglycemic patients [9]. The presence of osmotically inactive components, such as ethanol, on the other hand, will elevate serum osmolality but has been shown to have negligible effects on the reported serum sodium due to ethanol being an ineffective osmole [10,11]. In other words, the presence of ethanol leads to a hyperosmolar, but not hypertonic state. Thus, the reported serum sodium in a patient with alcohol ingestion can be trusted to be accurate, assuming the other scenarios discussed above have been excluded. However, if these nuances are not recognized, one can arrive at an erroneous etiologic classification of hyponatremia, and consequently erroneous management [8].

The serum osmolality is made up of many electrolytes (magnesium, potassium, calcium, etc.) and organic molecules (albumin, bilirubin, immunoglobulins, etc.) However, when estimating a patient’s serum osmolality, we commonly only consider a few of these components as seen in the following equation:

Equation 1: Calculated serum osmolality = 2 (Sodium) + (Blood Urea Nitrogen)/2.8 + (Glucose)/18

Equation 1 only uses sodium concentration, blood urea nitrogen (BUN) concentration, and glucose concentration. The reason for leaving out other electrolytes/compounds is that they collectively contribute to a small amount of the total osmolality compared to the electrolytes/compounds included in Equation 1. It can be appreciated from Equation 1 that sodium is the predominant extracellular electrolyte and contributes significantly to serum osmolality. As a result, in the setting of a true hyponatremia, we would anticipate the measured serum osmolality to be low (<285). However, the presence of osmoles not mentioned in Equation 1 (i.e., unmeasured osmoles) can contribute to the measured serum osmolality and produce a higher-than-anticipated result. In this scenario, a common calculation often used to assess the presence of unmeasured osmoles in the serum is the osmolar gap which is the difference between the measured osmolality and the calculated osmolality. A suggested normal osmolar gap ranges from -14 to +10, and if the osmolar gap exceeds this value, then the suspicion for an unmeasured osmole should be raised and investigations should be pursued for substances that elevate measured serum osmolality, such as toxic alcohols, and sugars (mannitol, sorbitol) [12]. Severe hyperlipidemia and hyperproteinemia could lead to pseudohyponatremia as explained above, which in turn can lead to a falsely low calculated serum osmolality (due to underestimation of serum sodium), and lead to a falsely elevated osmolar gap with a normal measured serum osmolality. Our patient had a consistently elevated serum osmolality which was an unanticipated finding, and other common etiologies of hypertonic hyponatremia pseudo-hyponatremia were ruled out.

When ethanol is detected in a patient’s serum at the time of assessing hyponatremia, one can divide the ethanol concentration by its molecular weight to assess the osmotic contribution. This can be added to Equation 1 to yield Equation 2:

Equation 2: Calculated serum osmolality = 2 (Sodium) + (BUN)/2.8 + (Glucose)/18 + (Ethanol)/4.6

If the osmolar gap corrects to the normal range after applying the correction for alcohol, as demonstrated in Equation 2 then the clinician can have a fair degree of confidence that there are no other unmeasured osmoles in the serum. In the context of hyponatremia, one can subtract the osmolar contribution of ethanol from the measured osmolality to get an idea of serum osmolality in the absence of ethanol. This could provide further insight into whether the serum osmolality would correct to a hypotonic range which would be anticipated in the setting of hyponatremia. If a urine ethanol concentration is obtained, this same process can be performed with urine osmolality which would yield more dilute urine as anticipated in the setting of hyponatremia [13].

In our patient, when the ethanol correction was applied to measured serum osmolality as above, the patient’s new measured osmolality fell within the hypotonic range as seen in Table 1. A small percent of serum alcohol is excreted in the urine and if a urine ethanol concentration were obtained, we anticipate that correcting for ethanol’s osmotic contribution would have yielded a more dilute urine [13].

## Conclusions

The evaluation of hyponatremia involves an assessment of the patient’s volume status along with additional serum and urine studies which can be compromised in various clinical settings leading to inappropriate conclusions and management. If the urine and serum studies, in this case, were taken at face value, would have led to erroneous management in the form of volume resuscitation or diuresis. In reality, because we determined that the elevation in serum and urine osmolality was related to alcohol, we were able to confidently diagnose him with beer potomania and recommend alcohol abstinence for management.

## References

[1] Adrogué HJ, Tucker BM, Madias NE (2022). Diagnosis and management of hyponatremia: a review. JAMA.

[2] Decaux G (2006). Is asymptomatic hyponatremia really asymptomatic?. Am J Med.

[3] Datta SK, Chopra P (2022). Interference in ion-selective electrodes due to proteins and lipids. J Appl Lab Med.

[4] Fortgens P, Pillay TS (2011). Pseudohyponatremia revisited: a modern-day pitfall. Arch Pathol Lab Med.

[5] Chopra P, Datta SK (2020). Discrepancies in electrolyte measurements by direct and indirect ion selective electrodes due to interferences by proteins and lipids. J Lab Physicians.

[6] Barkhuizen M, Hoffmann M, Zöllner EW, Erasmus RT, Zemlin AE (2019). Case report: an index of suspicion in hyponatraemia. Biochem Med (Zagreb).

[7] Dimeski G, Mollee P, Carter A (2006). Effects of hyperlipidemia on plasma sodium, potassium, and chloride measurements by an indirect ion-selective electrode measuring system. Clin Chem.

[8] Larkins MC, Zubair M, Thombare A (2024). Osmometer. StatPearls.

[9] Liamis G, Liberopoulos E, Barkas F, Elisaf M (2014). Diabetes mellitus and electrolyte disorders. World J Clin Cases.

[10] Oster JR, Singer I (1999). Hyponatremia, hyposmolality, and hypotonicity: tables and fables. Arch Intern Med.

[11] Mahboob T, Haleem MA (1988). Effect of ethanol on serum electrolytes and osmolality. Life Sci.

[12] Hoffman RS, Smilkstein MJ, Howland MA, Goldfrank LR (1993). Osmol gaps revisited: normal values and limitations. J Toxicol Clin Toxicol.

[13] Magner PO, Ethier JH, Kamel KS, Halperin ML (1991). Interpretation of the urine osmolality: the role of ethanol and the rate of excretion of osmoles. Clin Invest Med.

